# Multiple Coronary Artery Microfistulas in a Girl with Kleefstra Syndrome

**DOI:** 10.1155/2016/3056053

**Published:** 2016-04-30

**Authors:** Euthymia Vargiami, Athina Ververi, Hamda Al-Mutawa, Georgia Gioula, Spyridon Gerou, Fotios Rouvalis, Marios Kambouris, Dimitrios I. Zafeiriou

**Affiliations:** ^1^1st Department of Pediatrics, Aristotle University of Thessaloniki, 54642 Thessaloniki, Greece; ^2^Shafallah Medical Genetics Center, Doha, Qatar; ^3^2nd Department of Microbiology, Aristotle University of Thessaloniki, 54124 Thessaloniki, Greece; ^4^Pathology-Genetics, Sidra Medical & Research Center, Doha, Qatar

## Abstract

Kleefstra syndrome is characterized by hypotonia, developmental delay, dysmorphic features, congenital heart defects, and so forth. It is caused by 9q34.3 microdeletions or* EHMT1* mutations. Herein a 20-month-old girl with Kleefstra syndrome, due to a de novo subterminal deletion, is described. She exhibits a rare and complex cardiopathy, encompassing multiple coronary artery microfistulas, VSD/ASD, and PFO.

## 1. Introduction 

Kleefstra syndrome (KS) is a recently identified entity characterized by hypotonia, developmental delay, and distinctive facial appearance. Other common features include congenital heart and urogenital defects, epilepsy, microcephaly, and behavioral abnormalities. KS is caused by either a microdeletion of 9q34.3 or a mutation in the euchromatin histone methyltransferase 1* (EHMT1)* gene in the same region. Deletions account for 80% of cases and range from 0.04 Mb to cytogenetically visible abnormalities [1]. The critical region of the syndrome encompasses* EHMT1*, which is reported as the culprit gene for the syndrome [2, 3]. The prevalence of KS is still unknown; however, more than 100 individuals have already been described. Despite the wide variety in patients' deletion size, phenotype-genotype correlations are still weak, providing extra support for the major role of* EHMT1*.

Herein a 20-month-old girl with KS, due to a subterminal de novo deletion, is described. Although her phenotype is highly typical of KS, she exhibits a rather rare and complex cardiopathy with multiple coronary artery microfistulas, ventricular/atrial septal defects, and patent ductus arteriosus. Coronary anomalies have not been previously described in KS. The girl's clinical presentation, as well as the possible mechanism underlying her cardiac defect, is discussed.

## 2. Case Presentation

The patient is the only child of healthy, nonconsanguineous parents of Caucasian origin. Pregnancy was complicated by high rupture of membranes 8 weeks prior to delivery. She was delivered at 37 weeks by cesarean section, due to mother's past history of spinal surgery. During the 4th hour of life she developed transient tachypnea and was admitted to the NICU. On the 5th day she manifested poor feeding. She was discharged after 2 weeks in good condition.

**Figure 1:**
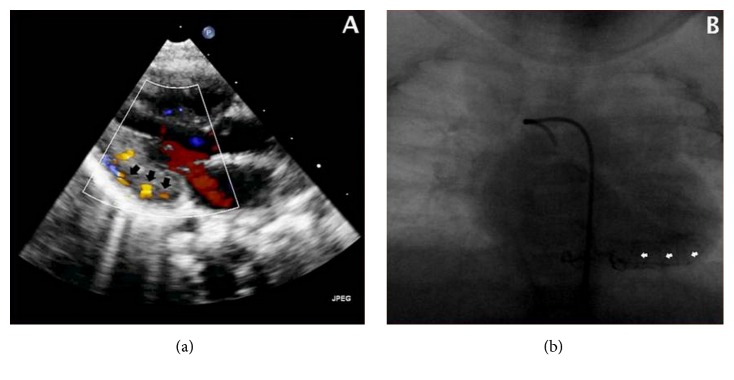
Multiple coronary artery microfistulas, originating from the left main coronary artery and draining into the left ventricle, as depicted in echo (a) and angiogram (b) (black and white arrows, resp.).

Regular pediatric follow-up at three months revealed a 3/6 systolic heart murmur. Subsequent echocardiography demonstrated multiple coronary artery microfistulas, originating from the left main coronary artery and draining into the left ventricle (Figure 1(a)). The latter was marginally hypertrophic but preserved normal contractility.

The left coronary artery was mildly dilated with an ostial diameter of 3.8 mm. Additionally, the patient manifested multiple small ventricular septal defects, as well as haemodynamically unsignificant patent ductus arteriosus and patent foramen ovale. The valves were normal. An angiogram performed at 4 months of age confirmed the above findings (Figure 1(b)).

Conclusively, the patient exhibited a complex cardiopathy, encompassing multiple coronary artery microfistulas, mild dilatation of the left coronary artery, ventricular and atrial septal defects, and patent ductus arteriosus. Nevertheless, she was asymptomatic and required no pharmaceutical or interventional therapy.

**Figure 2:**
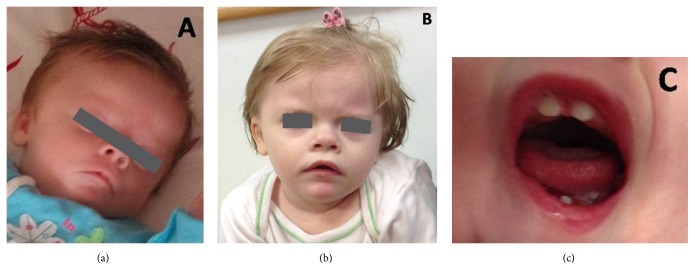
Facial profile of the patient at the age of 20 days (a) and 20 months (b); teeth anomalies at the age of 20 months (c).

Apart from the cardiopathy, additional clinical findings included dysmorphic features (hypertelorism, mid-face hypoplasia, short nose with upturned nostrils, tented upper lip, downturned corners of mouth, high arched palate, teeth anomalies, and clinodactyly of the 4th and 5th toes bilaterally), hypotonia, significant skin laxity, joint hypermobility, psychomotor delay, and progressive microcephaly-brachycephaly since the age of 4 months (Figure 2). Due to the constellation of findings, molecular SNP karyotyping was performed, using Illumina Human OmniExp-12 v2 BeadChips microarray technology at a median resolution of 4 Kb. The analysis revealed a microdeletion of 0.55 Mb on chromosome 9q34.3 (139.518.965-141.066.491), encompassing the critical region for KS. Parental analysis confirmed the de novo origin of the deletion.

Subsequent investigation at the age of 10 months included brain MRI, electroencephalogram, and auditory/visual evoked potentials, which were all normal. Due to the presence of microcephaly, the patient underwent cranial 3D-CT, which revealed fusion of both coronal sutures. At the age of 14 months she had an episode of generalized tonic-clinic seizures, followed by paroxysmal activity on postictal electroencephalograms. She was started on valproate and has been so far free of episodes.

At the age of 20 months, the patient has moderate-severe psychomotor delay with hypotonia, poor visual and auditory responsiveness, and a limited vocabulary of 1-2 words. She has moderate head control and poor trunk control but can sit with support. Her head circumference is below the 3rd centile (−2.5 SD) and her weight and height are at the 97th and 75th centiles, respectively. She is having physiotherapy and occupational therapy. As far as her cardiopathy is concerned, the condition remains asymptomatic with the majority of microfistulas gradually resolving in a spontaneous way.

## 3. Discussion

A 20-month-old girl with KS and an atypical cardiac manifestation with coronary artery microfistulas is herein reported.

Coronary artery microfistulas have not been so far documented in either 9q34.3 deletions or* EHMT1* mutations, despite the high prevalence of cardiac manifestations in the syndrome. In a cohort of 112 patients described by Willemsen et al. [1], approximately 40% manifested heart defects, mainly structural anomalies, such as ventricular and atrial septal defects, patent foramen ovale, valve and ductus arteriosus anomalies, tetralogy of Fallot, and so forth. More rare manifestations included an aberrant muscle band in the left ventricle of one individual, as well as cardiac arrhythmias in another two patients [1]. The clinical features of the cohort are summarized in Table 1.

Coronary artery fistulas (CAFs) are rare vascular anomalies presenting in 0.002% of the general population and involving either the great vessels or the cardiac chambers, mainly the right ones. Only 10% of CAFs affect the left heart structures [4], as in the index patient. CAFs are usually congenital in origin, probably resulting from the persistence of embryonic intertrabecular spaces and sinusoids [5]. Chemotactic, haemodynamic, and chemical factors have been associated with CAFs development [6], although their exact etiology remains still elusive. No genetic cause has been so far related to CAFs. There is one report of CAFs in dizygotic twins, but the authors suggested the presence of a poorly identified environmental factor during pregnancy [7].

As far as CAFs association with genetic syndromes is concerned, CAFs have been sporadically described in an infant with 22q11 deletion syndrome [8] and a man with mosaic Klinefelter syndrome [9], as well as three older patients with clinical diagnoses of Marfan [10], Goldenhar [11], and Rendu-Osler-Weber [12] syndromes. The latter condition is associated with 5 different genetic causes, including* ENG* mutations at 9q34.11 [13], which is 9 Mb away from the critical Kleefstra region. Moreover, molecular analysis was not undertaken in that patient and, thus, neither the diagnosis nor the exact genetic type of Rendu-Osler-Weber was confirmed. The infant with 22q11 deletion manifested left ventricular noncompaction and a CAF between the left coronary artery and the right ventricle outflow tract [8], whereas the man with Klinefelter syndrome had a CAF between the left coronary artery and the right ventricle [9]. The patient with Marfan syndrome had a left CAF draining into the superior vena cava and the right superior pulmonary vein, as well as a second fistulous communication between the left coronary sinus and both atria [10]. Marfan syndrome is associated with mutations or deletions in* FBN1* at 15q21.1 [14], but the index patient did not have molecular confirmation of the diagnosis. Last but not least, a CAF between the left coronary artery and the right atrium was reported in a woman with Goldenhar syndrome [11]. Goldenhar syndrome has no unique heritable cause but has been associated with chromosomal abnormalities, including pericentric inversion of chromosome 9 [46 XY inv 9 (p11; q13)] [15]. It should be noted that none of the above syndromes involve 9q34.3 deletion. Moreover, all five patients demonstrated solitary fistulous communications, in contrary to the multiple microfistulas of our patient.

The deleted region in the index patient contains 38 OMIM genes. Seven of them are disease-causing:* AGPAT2, MAN1B1, GRIN1, TPRN, SLC34A3, NSMF*, and* EHMT1*. Apart from* EHMT1*, the other six are associated with congenital lipodystrophy type 1, mental retardation, deafness, hypophosphatemic rickets, and hypogonadotropic hypogonadism. Due to their high haploinsufficiency scores (>60%) [16],* AGPAT2, TPRN, SLC34A3*, and* NSMF* are highly unlikely to contribute to the patient's phenotype.* MAN1B1* and* GRIN1*, which are both associated with intellectual deficits have low indexes of 30.3% and 33.4%, respectively [16]. However, neither these, nor the rest of the 38 deleted genes, have been related to coronary abnormalities so far.

Heart anomalies in KS are equally present in patients carrying a 9q34.3 deletion or an intragenic* EHMT1* mutation [1].* EHMT1* deficiency is, therefore, strongly implicated in the cardiac phenotype of the syndrome. It should be noted, however, that two patients with* MAN1B1* mutations exhibited dilatation of the aortic root and ventricular septal defect, respectively, along with mental retardation, hypotonia, mild dysmorphic features, joint hypermobility, and skin laxity. The latter feature was prominent in our patient, albeit not typical of KS. It is also noteworthy that neither these patients nor ours manifested disruption of* COL5A1* at 9q34.3, which is the culprit gene for classic Ehlers-Danlos syndrome and, also, linked to the hypermobility in KS.* MAN1B1* deficiency is currently associated with autosomal recessive intellectual disability. Nevertheless, the two patients were unresolved cases of congenital disorders of glycosylation (CDG) type II and were found to carry* MAN1B1* mutations through exome sequencing. Five other patients with CDG II and* MAN1B1* mutations have been so far identified. All patients carried either homozygous or compound heterozygous mutations [17]. Heart involvement has been often described in patients with CDG, ranging from cardiomyopathy to conotruncal defects [18]. Both manifestations have been recently studied in mouse models of the disease [19, 20]. One proposed mechanism for structural heart defects involves deletion of N-cadherin in neural crest cells, which, subsequently, fail to participate in embryonic cardiac development [19].

Unfortunately, no experimental animal model for either CAFs or cardiac manifestations in KS has yet been developed. An EHMT1^+/−^ mouse model has only recently recapitulated the developmental features of the core KS phenotype [21]. Future experimental work with animal models, as well as identification of new patients with KS, will probably dissect the cardiac features and identify the culprit gene, whether it is* EHMT1* alone or* EHMT1* in conjunction with* MAN1B1* and others. As far as CAFs are concerned, this is their first description in KS and, thus, a definitive link cannot be established. It can be, however, supported that cardiac manifestations, either common or rare, warrant KS investigation in unresolved cases with syndrome-indicative phenotypes.

## Figures and Tables

**Table 1:** Summary of main features in 91 reported patients with 9q34.3 deletion [1] and in the index patient.

Clinical features	% in 91 patients	Present in proband
Growth parameters		
High birth weight	9	−
Microcephaly	50	+
Short stature	32	−
Overweight (BMI > 25)	28	−
DD/ID	100	+
Heart defect	41	+
Renal anomaly (including vesicoureteral reflux)	12	−
Behavioral/psychiatric problems (including autistic features, attention deficit problems, self-mutilation, aggressive and emotional outbursts/crises, and severe sleep disturbance)	54%	−
Recurrent infections	26	−
Hearing deficit	23	−
Gastroesophageal reflux	19	−
Epilepsy	36	+
Anomalies on brain imaging	58	−
Musculoskeletal anomalies (including joint hypermobility, scoliosis, and club foot)	25	+
Teeth anomalies	<10%	+
Hyperelastic skin	0	+
